# External beam radiation therapy or high intensity focused ultrasound for localized prostate cancer: a matched pair analysis in the prostate-specific antigen era

**DOI:** 10.1186/2050-5736-3-S1-O55

**Published:** 2015-06-30

**Authors:** Albert Gelet, Sebastien Crouzet, Olivier Rouviere, Jean-Yves Chapelon, Murielle Rabilloud

**Affiliations:** 1 Edouard Herriot Hospital, Lyon, France; 2 Hospices Civils de Lyon, Lyon, France; 3 Inserm, Lyon, France

## Background/introduction

Background: In the absence of randomised study data institutional series have shown High Intensity Focused Ultrasound (HIFU) to produce excellent overall and cancer specific survival rates in patients with localized prostate cancer (LPCa) compared with alternative curative treatments.

Objectives: To evaluate the oncologic outcome of patients treated with HIFU *versus* conformal external beam radiation therapy (C-EBRT) without previous or associated androgen deprivation (AD). This study is designed to overcome limitations of case series studies by using a matched pair design in patients treated contemporaneously with HIFU and C-EBRT in two institutions in the same town.

## Methods

Design, setting and participants: 256 eligible patients with intermediate risk prostate cancer (d’Amico classification) treated between 2000 and 2005 were prospectively followed and matched to a 1:1 basis following know prognostic variables: prostate-specific antigen (PSA) level and Gleason score. 190 perfect matches of patients (95 in each group) were further analysed. Progression free survival rate were the primary endpoint. Other endpoints were secondary used of salvage therapy, and survival rate without salvage palliative androgen deprivation therapy (S-ADT). The progression free survival rates were calculated with Kaplan-Meier estimate. For progression free calculation, failure was defined using the Phoenix definition (nadir + 2ng/ml) or at the time of a salvage treatment for local relapse evidenced by control biopsy.

## Results and conclusions

Results: The seven years progression free survival rate was not significantly different after HIFU than after C-EBRT (47% *versus* 52%, p: 0.311). The palliative androgen deprivation free rate at seven years was significantly different after HIFU than after C-EBRT (85% *versus* 58%, p: 0.002).

Conclusion: The progression free survival rate was not significantly different after HIFU use than after C-EBRT but the rate of patients who need palliative S-ADT was significantly different after HIFU or C-EBRT: Higher rate of S-ADT was associated with C-EBRT use than with HIFU use.

